# Wet market biosecurity reform: Three social narratives influence stakeholder responses in Vietnam, Kenya, and the Philippines

**DOI:** 10.1371/journal.pgph.0001704

**Published:** 2023-09-06

**Authors:** Kevin Bardosh, Renzo R. Guinto, Salome A. Bukachi, Tran Minh Hang, Marianne K. Bongcac, Mara Ysabella M. de los Santos, Caroline M. Mburu, Jackielyn Abela, David Kelly, Cecily Maller

**Affiliations:** 1 School of Public Health, University of Washington, Seattle, Washington, United States of America; 2 Edinburgh Medical School, University of Edinburgh, Edinburgh, United Kingdom; 3 Centre for Urban Research, Royal Melbourne Institute of Technology, Melbourne, Australia; 4 Planetary and Global Health Program, St. Luke’s Medical Center College of Medicine-William H. Quasha Memorial, Quezon City, Philippines; 5 Institute of Anthropology, Gender and African Studies, University of Nairobi, Nairobi’, Kenya; 6 Institute of Anthropology, Vietnam Academy of Social Sciences, Hanoi, Vietnam; 7 Department of Social Anthropology, University of St Andrews, St Andrews, United Kingdom; 8 Palawan State University, Puerto Princesa, Palawan, Philippines; Keele University, UNITED KINGDOM

## Abstract

In 2020, Covid-19 led to global policy statements promoting bans and reforms to wet markets in Asia and Africa to prevent future pandemics. We conducted a comparative, exploratory qualitative study in 2021 in three countries (Kenya, Vietnam and the Philippines) to understand the social and political dimensions to biosecurity reform at wet markets. This included 60 key informant interviews and rapid ethnographic research in 15 markets, as well as a review of policy documents and online media articles. We found no evidence that the rhetoric of pandemic spillover that emerged in 2020 had any influence on policy or reform efforts apart from those related to Covid-19 infection control. Rather, we identified three main narratives that frame the problem of biosecurity and preferences for reform. The first, a *human health narrative*, questioned global framings about pandemic risk, viewed markets as sources for food security rather than disease, emphasized the need to strengthen the control of endemic diseases, and conceptualized health through the lens of ‘freshness’ rather than biomedical categories. A second *modernization* narrative approached biosecurity as part of a broader process of socio-economic development that emphasized infrastructural gaps, spatial arrangements, cleanliness and a conflict between reform and economic interests. A third narrative centered on *local livelihoods* and the tension between local market stakeholders and biosecurity and modernization efforts. This final narrative called into question the appropriateness of certain regulations and policies, including bans and closures, emphasized the importance of preserving cultural heritage and highlighted the need for collective political action to resist certain veterinary policies. In conclusion, wet market biosecurity strategies occur in the context of three contrasting narratives that emphasize different aspects of health and risk, and reflect different worldviews and interests. Within this context, there is a need for local government to strengthen market management and biosecurity in ways that enhance the agency of market stakeholders and strengthen local livelihoods and food security as part of a pluralistic and democratic politics.

## Introduction

The Covid-19 pandemic reinstated concerns about the potential role of wet markets in amplifying emerging disease spillover events. Wet markets in Asia were singled out in 2020 as an alleged source of Covid-19 and future global pandemic risk with calls to close, ban, regulate, and reform them [1,2]. The concerns centered heavily on wild animals in so-called ‘wildlife wet markets.’ This led to high-level political pressure for veterinary public health and biosecurity reforms including by the Australian Minister of Agriculture at a G20 meeting in April 2020 [3]. In these narratives, pandemic risks are frequently linked to wider concerns about biological and ecological conservation, the global wildlife trade, and animal welfare and animal rights [4,5].

However many wet markets do not sell wildlife or bushmeat; in fact, wildlife represent only a small fraction of the animals and meat sold at wet markets in Asia and Africa [6]. There are a diverse range of “wet markets” that need to be distinguished by their scale, produce and type of animals, among other factors. We define ‘wet market’ broadly to mean any fresh-food market where live animals (poultry, ruminants, seafood and wildlife) are kept, slaughtered and sold to consumers alongside fruits, vegetables and/or grains. In this sense, many “wet markets” are synonymous with “traditional” or “fresh-food” markets [7,8]. While emerging diseases with pandemic potential represent low probability but high impact events, the risk of endemic food-borne and zoonotic diseases transmission at wet markets (*Salmonella*, *Campylobacter*, *E*. *coli*, etc.) contribute to more frequent and significant local disease outbreaks [8–10]. Furthermore, the framing of wet markets as sources of disease and threats to biodiversity protection may generate simplistic and punitive policies that ignore positive contributions to local nutrition, livelihoods, and sociocultural wellbeing [7,11]. This may have unintended negative socioeconomic, health and wellbeing consequences for communities and the resilience of food systems.

This exploratory qualitative research project explored biosecurity practices and reform at wet markets during the Covid-19 pandemic across three case studies in Vietnam, Kenya and the Philippines. These countries were chosen to represent a range of wet market systems and government responses to the pandemic. Our primary aim was to explore how the global rhetoric of wet market bans and biosecurity reforms have been translated into local settings and impacted communities, food landscapes and public health responses.

## Methods

A research team based in each of the three countries conducted the data collection and analysis within that country. Mixed-method social research was conducted from June-December 2021 in three phases: 1) policy and media analysis, 2) key informant interviews, and 3) rapid ethnography. Research tools used by each team are provided in S1 and S2 Files.

### Policy and media analysis

In each country, we first collected online national policy documents and online news articles related to wet markets and the wildlife trade over the last five years using a search term rubric (Table 1). This included laws, policies, infection control guidelines, and official documents related to the wildlife trade, etc. We also reviewed the academic literature for publications of relevance for each country. We synthesized key insights from these sources into preliminary country reports, which are not presented in this paper.

**Table 1:** Policy and media analysis sample.

	Kenya	Philippines	Vietnam
Policy documents	18	29	9
Online media articles	5	38	126

### Key informant interviews

**Fig 1:**
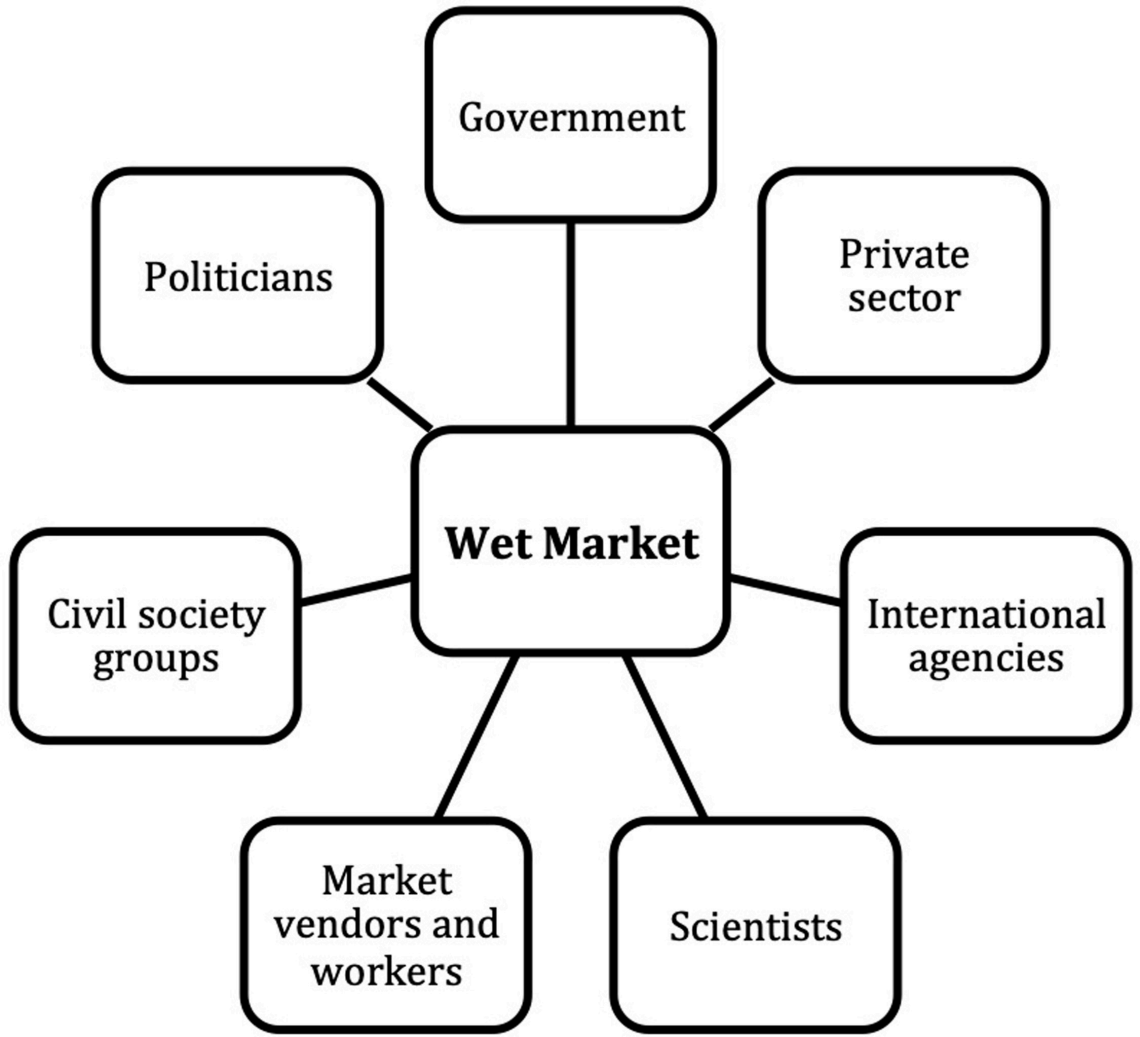
Stakeholder map.

From the policy and media analysis, we created a list of 30–50 key stakeholders in each country. We identified 7 main stakeholder groups that influence wet market biosecurity (**Fig 1**). Categories were similar across the countries, although specific organizations were different (see S4–S6 Files). Each country team produced a stakeholder map.

We aimed to interview a representative sample of stakeholders. Maps were used as the basis for narrowing down the final selection of key informants to 20 individuals per country (Table 2). Where certain informants were not available, substitutes were found, as is consistent with a purposive sampling approach. Market managers, regulators, vendors and workers as well as politicians were not included in the key informant interviews but were part of the subsequent rapid ethnographic fieldwork. Interviews lasted roughly 1 hour each and were conducted in-person or remotely depending on pandemic restrictions. Interviews were both audio-recorded and summarized (Philippines) and documented through hand-notes (Kenya, Vietnam).

**Table 2:** Selected key informants per country.

	Kenya	Philippines	Vietnam
Government	**8**	**11**	**5**
Scientist	**6**	**2**	**3**
International agency	**5**	**4**	**3**
Private sector	**1**	**1**	**5**
Civil society	**0**	**2**	**4**
**Total**	**20**	**20**	**20**

### Rapid ethnography

The final phase of the project (October-December 2021) involved visiting a purposive sample of markets in each country for 1–2 days per market and using rapid ethnographic methods (observations, informal group interviews, informal interviews) to understand the opinions and experiences of market workers, vendors, managers and customers. Ethnographic research involved 1–3 social scientists visiting the market for the duration of the day. Observational notes and photographs were taken of the physical condition of the market, infrastructure, trading and transportation, hygiene, inspections and biosecurity practices. Key individuals with an important role related to the operation of the market (e.g. market management board members, traders, transporters, slaughterhouse workers, veterinarians, cleaners) as well as customers were selected for interviews and group discussions. Question templates were created prior to fieldwork to guide these conversations. Data collection consisted of hand-written notes and photographs; audio recording was not considered appropriate for informal interviews.

To select the markets, we first developed a characteristics rubric adapted from Lin et al.’s [6] classification of wet markets based on potential disease risk. This included whether the market sold dead/live animals, wild/domesticated animals, and/or perishable/non-perishable goods. We also accounted for the type and age of the market, geography (urban/rural, coastal/inland), ethno-linguistic and socioeconomic groups that are known to frequent the market as well as historical knowledge and local reputation (cleanest wet market, central hub for wildlife trade, etc.). Using this rubric, each country generated a list of 10 markets with diverse characteristics, with the initial goal of purposively selecting 2–3 per country that would account for key variations common in the country.

In total, we visited 15 markets over 1–2 days each in Kenya (5) and the Philippines (2) and 3–5 days each in Vietnam (8) (Table 3). In Kenya, we selected two of the largest animal markets in Nairobi and three rural markets in Busia County, a cross-border area on the Kenyan-Ugandan border. In Vietnam, we selected 8 wet markets in greater Hanoi, representing a range of wholesale, retailer and temporary markets. Unfortunately, it was not possible to travel outside Hanoi at this time to rural locations due to Covid-19 restrictions. In the Philippines, we chose one urban site in Manila and one rural market in Palawan Island, a remote biodiversity hotspot in the country. Unless stated otherwise below, each market sold live/dead animals and animal parts, conducted some animal slaughter, and sold an assortment of fresh hot food, vegetables, fruits, grains and various consumer goods.

**Table 3:** Wet markets included in the study.

	Kenya	Philippines	Vietnam
**Urban**	1. Nairobi Meat Market	1. Arranque Market, Manila	1. Long Bien 2. Ha Vy 3. Hai Boi 4. Den Lu 5. Ha Dong 6. Dong Xuan 7. Nghia Tan 8. Bo Song (All in greater Hanoi)
	2. Local Terminal Market 1- Nairobi		
**Rural**	3. Local Terminal Market 1 –Busia	2. Rio Tuba Market, Palawan Island	
	4. Fish Market–Busia		
	5. Rural Local Terminal Market 2- Busia		

* Fieldwork in Kenya also included visits to a rural pig slaughterhouse and 3 different rural butcheries in Busia County.

### Data analysis

**Fig 2:**
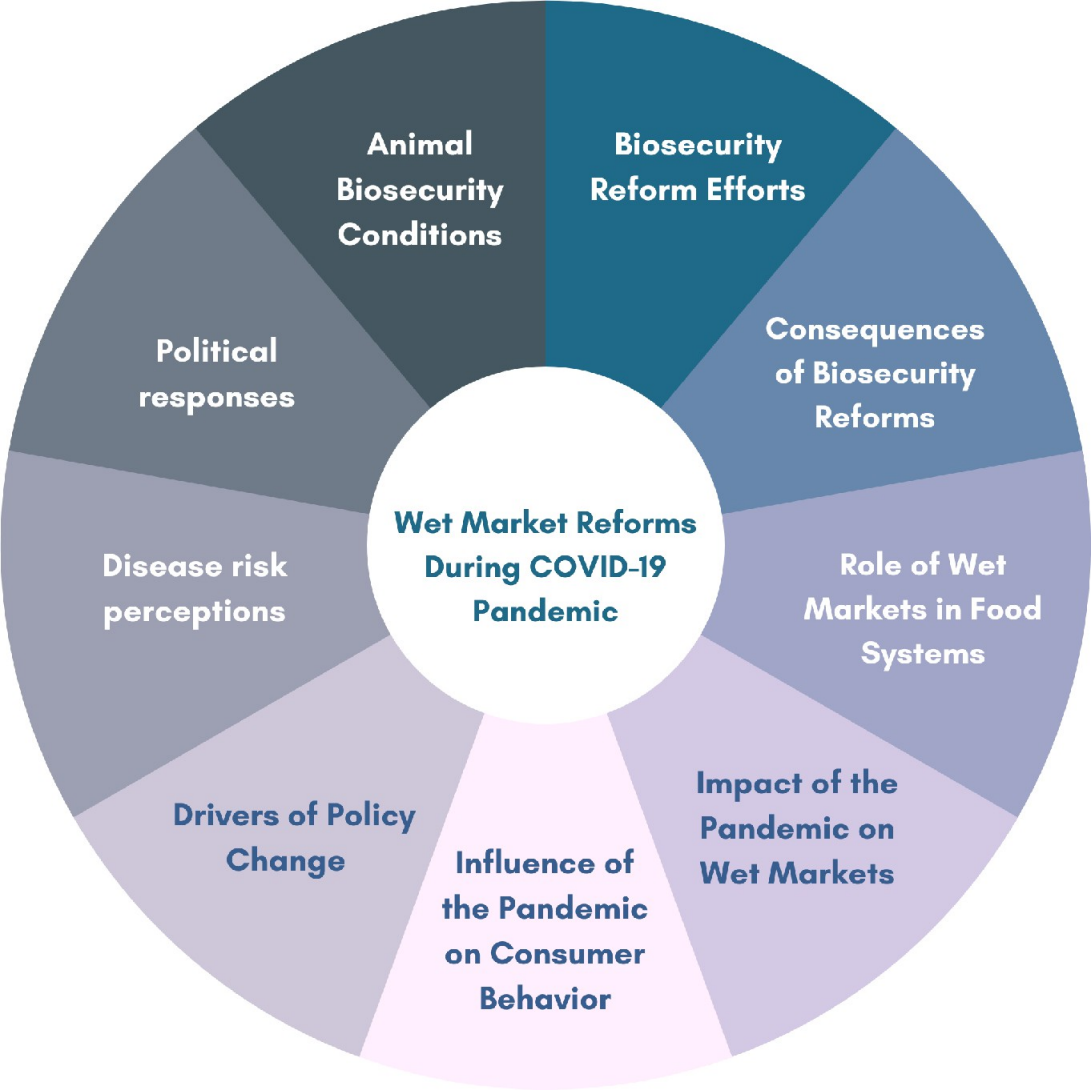
Ten main themes used in the final analysis of fieldwork data.

Each country team conducted thematic analysis of the interview and rapid ethnographic data they collected using written accounts or summaries of interviews, combined with the policy and media analysis. Photographs from the rapid ethnography were used for illustrative purposes. The analysis was collated into a country report. Prior to drafting this report, biweekly online team meetings allowed us to discuss emerging findings. The team agreed to a generic thematic structure to the country reports to assist with comparative analysis. We identified 10 main themes to guide the final analysis of fieldwork data, summarized in **Fig 2**. In general, these emerged from the question guides. Country reports were collated and analyzed comparatively; multiple rounds of review and analysis were undertaken by each country team, before being consolidated by the first author.

### Ethical clearance and participant consent

Ethical clearance was obtained from RMIT University (2021-24245-15509) (Australia), St. Luke’s Medical Center (SL-21126) (Philippines) and the Kenyatta National Hospital/University of Nairobi Ethical Review Committee (KNH-ERC/A/322) (Kenya). The research was deemed exempt in Vietnam (social research in Vietnam does not require ethics approval; however, the Vietnamese data collection was covered by the RMIT ethics approval).

An invitation letter was sent to each key informant prior to interviews, and consent was obtained by email and verbally before the interview. Permission for the rapid ethnographic research was obtained from the appropriate government and/or private sector authority before fieldwork began at each market site. Verbal consent was obtained for informal group and individual interviews.

## Results

**Fig 3:**
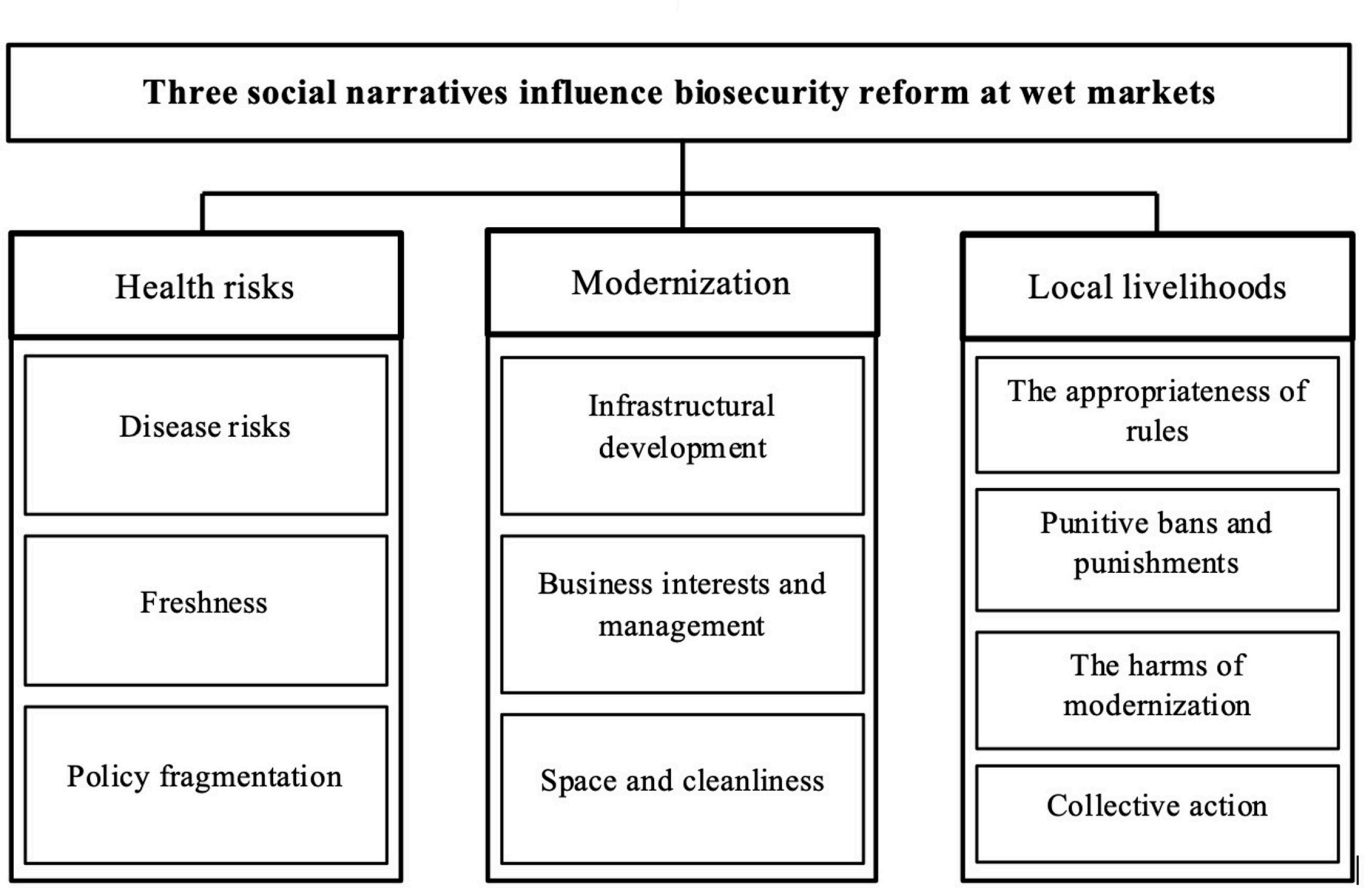
Conceptual framework.

Our analysis identified three main narratives about wet markets that shaped how different stakeholders perceived and engaged with biosecurity reforms (see **Fig 3**): 1) health risks, biosecurity and food safety, 2) economic interests and the drive towards modernization and 3) local livelihoods, restrictions and politics. These narratives were emphasized and conceptualized differently by different stakeholder groups.

## Narrative 1: Health risk, biosecurity and food safety

The first narrative framed wet markets through the lens of ‘*health’*, which consisted of contested notions of risk, biosecurity and food safety.

### Wet markets, Covid-19 and pandemic spillover

We found no evidence that global rhetoric, in 2020, to ban wet markets and restrict wildlife sales influenced any market reforms in the case study countries in 2020–21. Rather, policy was quickly subsumed by rising infections and the challenges of lockdown and other non-pharmaceutical interventions; the only pandemic policies that influenced wet markets related to infection control.

Despite the alleged market origin of Covid-19 that dominated media in 2020–21, wet markets were not perceived as sources of emerging pathogens. Rather, they were overwhelmingly viewed as essential means of providing food security and livelihoods–of *improving* health–rather than ‘risky’ or unhealthy places. Participants voiced concerns about the representation of markets as “petri dishes” for novel diseases by international media. The pandemic led to a range of temporary biosecurity measures at wet markets that were not aimed at preventing novel pathogen spillover but in containing SARS-CoV-2 (Table 4). Government officials and scientists believed that the media and international organizations over-prioritize novel wildlife-related pandemic risks over food-borne diseases, epizootics (African Swine Fever and Highly Pathogenic Avian Influenza) and endemic zoonotic diseases (see Table 5).

**Table 4:** Pandemic restrictions at markets, identified in the case study countries.

Type of pandemic restriction	Interventions*
Behavioural	Mandatory face masks, face shields, and gloves; washing and disinfecting requirements; mandatory vaccination; no hand shaking; disinfecting sales areas; requiring customers to wash hands; no spitting; more frequent garbage collection.
Movement	Social distancing; curfews; transport restrictions; shopping voucher systems during lockdowns; greater spacing between market stalls; seat arrangements; limit visiting time; avoiding the area if sick; body temperature checks; testing requirements; designated entry and exit points.
Closure	Lockdowns; closures if COVID-19 cases are reported; closures if the market does not abide by the pandemic restriction rules.

* This table summarizes all of the various types of interventions used in the three case study countries.

**Table 5:** Past disease outbreak believed to have been associated with wet markets, according to key informants and local market stakeholders.

Kenya	Philippines	Vietnam
Avian Influenza, Rift Valley Fever, TB, brucellosis, measles, salmonella, E coli, cholera, typhoid, AMR.	Highly Pathogenic Avian Influenza (H5N6, H5N1), Foot-and-Mouth Disease (FMD), African Swine Fever (ASF), anthrax, canine parvovirus (CPV), *salmonella*, amoebiasis, cholera	H5N1, African Swine Fever, MERS-CoV, measles, salmonella, E coli, AMR.

* This list includes disease outbreaks reported by key informants in our interviews. In general, we found that market staff and vendors had a lack of awareness regarding these previous disease outbreaks and could not name specific diseases.

The lack of emerging pathogen risk was often framed in relation to a general perception that wild ‘exotic’ animals were rarely sold at formal markets. We found that wildlife rarely ends up at the formal wet markets we visited, although clandestine sale occurs in ‘black market’ or ‘shadow selling’ (Table 6), especially in informal settlements in Kenya, rural and remote markets in the Philippines, and unofficial mobile bird markets in Vietnam. Policy debates about wildlife meat were driven by concerns about ecological conservation and biodiversity protection, rather than emerging pathogens. This has generated awareness among consumers in all three countries that the indiscriminate trading of wild animals and wildlife is prohibited, although wildlife farming was acknowledged to be growing, fudging boundaries in current laws and oversight.

**Table 6:** Wildlife traded and sold at the selected research markets.

	Kenya	Vietnam	Philippines
Directly observed	None	Snakes, turtles, rats, birds, rabbits, cats	Birds (lovebirds, parakeets)
Mentioned by informants	Zebra, Impala, buffalo	Snakes, turtles, rats, birds, rabbits, cats, pangolin	Live wild animals: Exotic reptiles (pythons, turtles, monitor lizards, iguanas, geckos, chameleons) and exotic birds (Myna birds, cockatoos) Wildlife products: wild boar meat, turtle meat, pangolin meat

### Meat safety: Freshness & veterinary inspections

Concerns about human health risks at markets were related to meat safety, largely interpreted through the concept of ‘freshness.’ Meat is frequently touched by customers and sellers, and its visual color and texture are considered intrinsic to determining levels of freshness. In urban markets, slaughterhouse quality certification and timestamps functioned as determinants of freshness, while in rural markets this involved on-site slaughter and social connection or knowledge about the source of an animal. In some markets, incandescent bulbs have been replaced by LED lighting to enhance visual freshness. In general, frozen foods were viewed as “unfresh”, of lesser quality and safety.

*“Due to our culture*, *we have concentrated on fresh produce and we don’t like packaged and processed food*. *We are averse to labelled standardised foods*, *many consumers thus believe in these informal markets; they want meat from butcheries and to be able to point and say “give me that cut of meat*!*”* (KII, Veterinary officer, Kenya)

*“There’s trust for both ‘suki’—for the buyer and the seller*. *[…] There’s that trust that what you are buying is not double-dead*, *hot meat*, *rancid*, *spoiled*, *putrid*.*”* (KII, Labor Group Representative, Philippines)

*“I want to buy food in wet markets because the price is cheaper than in supermarkets*. *Buying food in wet market*, *I can choose ‘The fish is swimming*, *the chicken is crowing’ to make sure it fresh*. *The sellers also help me to to scale fish and pluck chicken feathers*. *Furthermore*, *the frozen food is not tasty*.” (Rapid ethnography, customer, Vietnam)

We observed many conditions classified according to biomedical standards as unhygienic or risky: touching meat with bare hands; adjusting masks or handling money without washing hands; mixing cooked and raw meat; meat stored on the ground; blood retrieved from drainage for sausages; use of stagnant water; lack of protective gear; meats left in prolonged sunshine, etc. Vendors did not think that diseases could be transferred by touching and interacting with the meat products on display at their stalls.

We found that the divide between the formal and informal sector can be difficult to determine; for example, according to one senior key informant, there are an estimated 749 registered slaughterhouses in Hanoi for poultry, pigs, and cow/buffalo but many are organized on the pavement, are seasonal (especially for poultry), operate during the night (from 11pm-6am) and are technically illegal since poultry are banned from the inner city. Even in larger markets, some traders actively slaughter on site, making food safety work challenging for veterinary inspectors. Market stakeholders were well aware of the gap between formal regulations and local conditions (Box 1).

**Box 1. Reluctance for photographs in markets, Kenya**

There was a lot of hesitancy in our taking photographs in Kenyan wet markets, especially in the slaughterhouses, perhaps because the meat inspectors and market managers were already aware of the poor conditions of the markets and negative publicity that has been portrayed in the media. These leaders noted that they were doing their best given the limited funds and weak infrastructure and they were afraid of the pictures being used in the media which would hurt their business.

The unscrupulous business practices of certain traders and sellers were viewed as “health risks” because they sold “unfresh meat.” In the Philippines, informants noted protocol violators tampering documents for interprovincial transport and the sale of frozen meat (traditionally prohibited in wet markets). In Kenya, this included the sale of chickens that died unexpectedly or from diseases, the use of cat meat in “*samosas”* (a kind of stuffed pastry) and the clandestine sale of wildlife meat, mostly sold in informal settlements. Some markets were known as “dumping grounds” for poor quality meat, often focused on the urban poor and lower socio-economic groups.

*“There have been cases of game meat sold in Nairobi and this often happens in the informal settlements where this meat is sold from the back of vehicles and is uninspected*. *Some unscrupulous business men have also been going around chicken farms and buying dead chickens which they slaughter and sell to poor consumers for Ksh 100 when chicken costs Ksh 500”*. (KII, Veterinarian, Kenya)

*“Because in the cities*, *in bigger areas*, *there is an inspector*. *The inspectors are trained*, *if not they’re veterinarians who actually say this is good for human consumption*. *But they don’t reach the hinterlands […] in Northern Palawan*, *they sell wild pig along the road*. *Like whatever they killed for instance that evening*, *they will sell it*. *So*, *there’s no control there*.*”* (KII, Researcher, Philippines)

*“Near industrial parks*, *there are often temporary markets on the roadway*. *Workers like to buy goods on the roadside quick and cheap*, *but are less concerned about food quality and safety*. *Many sellers pluck chicken and duck feathers right on the roadside*, *then pour wastewater and waste on the roadway*, *clogging drains with unbearable stench*.*”* (KII, Health official, Vietnam)

### One health and policy fragmentation

Government officials and scientists focused heavily on the need to address the fragmentation and lack of coordination of policy and regulation, which they highlighted as major barriers to wet market biosecurity improvements. Coordination challenges between different tiers of government in newly decentralized systems were repeatedly highlighted.

“*In the livestock industry there are more than 20 policies and now we are consolidating them into four policies*. *The first is the livestock bill*, *the animal health bill*, *the veterinary public health bill then the animal welfare and protection bill and they are in the process of becoming bills of parliament”*. (KII, Veterinary officer, Kenya)

*“In the whole country*, *there are 434 large slaughterhouses with veterinary control; 25*,*000 small slaughterhouses*, *of which only 30% have business licenses*, *70% do not have licenses*.*”* (KII, Government veterinarian, Vietnam)

*" The basis of our policies are good but when they’re already downstream*, *the difference in implementation*, *perception*, *as well as understanding of the policies* . . . *And somehow [local government] because of their autonomy…they can follow or not that national laws*.*"* (KII, Health official, Philippines)

Scientists called for improvements in “science-based” policies and regulation, and the need to better implement a One Health approach, although they also stressed the significant shortages in funding and the difficulties in translating research into improved policy. One Health national units have recently been established: The Philippine Committee on Zoonosis (PhilCZ) and Kenyan Zoonotic Disease Unit (ZDU), as well as national policy frameworks such as Vietnam’s One Health Partnership for Zoonoses (OHP). However key informants called for more *“authentic collaboration”* and maintained that challenges in collaboration include “*turfing*,” lack of funding for collaborative work, poor local ownership, and continued neglect of wildlife and environment approaches.

*“So*, *the committee on zoonosis that we attend to*, *together with the Department of Health*, *is a big step for recognizing […] whatever threat that is zoonotic in nature*. *Unfortunately*, *of course*, *[…] with the multitude of diseases*, *we are not always given enough funds to do either surveillance*, *testing or something*.*”* (KII, Government official, Philippines)

*“The agenda in wet markets is mainly guided by external forces such as the WHO but we need to find a voice for ourselves*. *A lot of research has been done by international groups but because these diseases are endemic with us*, *we need to make these diseases a priority*. *Food safety is not even addressed in our curriculum…We don’t know what our food safety problems are and food safety issues need to be prioritized and we should identify which value chains are at risk*. *We lack the capacity*, *lab*, *surveillance*, *policies*, *legislation and traceability systems”*. (KII, Researcher, Kenya)

*“Currently*, *in Vietnam*, *food is managed according to the Law on Food Safety*, *Article 61 stipulates "Responsibility for state management of food safety"*, *in which*, *the Ministry of Health has the main responsibility*, *other Ministries*, *organizations and local authorities must cooperate with the Ministry of Health*. *However*, *some regulations are still unclear*, *lack of specificity on the assignment of responsibilities among ministries*, *organizations and local authorities*.*”* (KII, Health official, Vietnam)

## Narrative 2: Economic interests and the drive towards modernization

Wet markets were viewed as physical sites that represented the socio-economic conditions of an area, and efforts to reform biosecurity were framed as part of a broader process of modernization. This was discussed not in the language of health but in reference to infrastructural improvement, cleanliness, and the negotiation between the formal and informal sector.

### Infrastructural improvements

**Fig 4:**
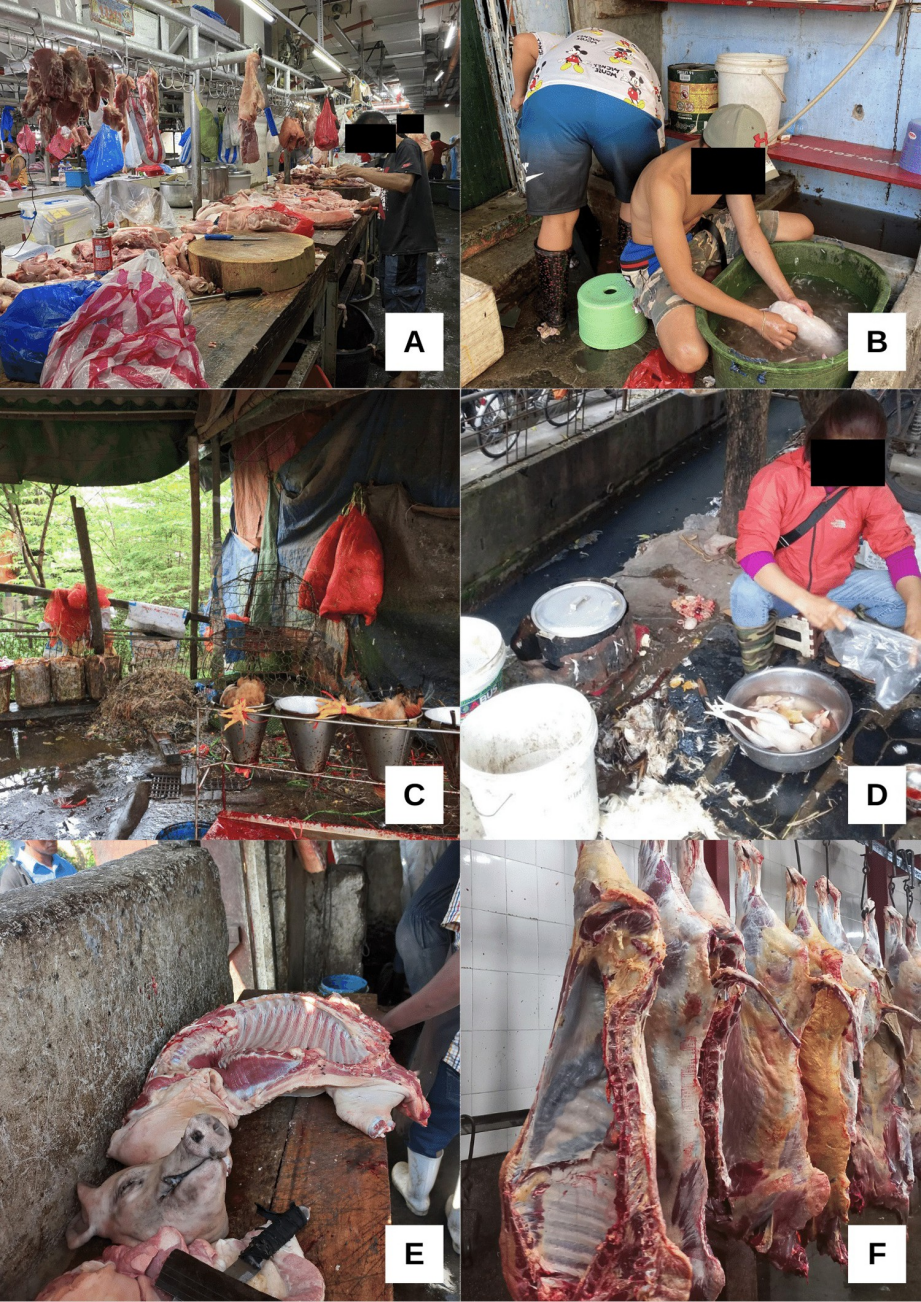
Slaughter practice photos at wet markets in the Philippines (A/B), Vietnam (C/D) and Kenya (E/F).

Gaps in biosecurity were explained by reference to the lack of physical infrastructure at wet markets (See **Fig 4**). Market vendors, workers and managers emphasized recent infrastructure improvements over the last few decades including better water and sanitation facilities and hygienic conditions in slaughter, transport, and waste collection.

Many infrastructure gaps still exist. We observed the unfiltered discharging of wastewater into sewers and rivers; crowded animal cages; a lack of water and garbage disposal; and inadequate disinfection and cleaning. In Vietnam, we often observed no area to safely dispose of animal parts, especially on-site slaughter for poultry and animals with signs of disease. In meat markets in Kenya, we noticed clogged drains, meat on the floor and disposal of animal waste on the open ground. In an urban market in Manila, Philippines, we found only three water faucets available at the meat section for usage; previously, each stall had its own faucet, but most were removed to cut down on water expenses at the market.

### Business interests and management

Economic forces were invoked to explain a lack of infrastructure and biosecurity, especially in Kenya. Biosecurity impinges on the profits of private sector actors and workers; at the same time, markets are a tax revenue source. Legal requirements demand that slaughterhouses and markets meet biosecurity standards, but the large capital investment needed minimizes the incentive to impose consequences for non-compliance. Market managers acknowledged that implementation and compliance with government policies was expensive and time consuming.

*“People perceive [that biosecurity investments] will take away profits but they need to know once we have them in place*, *they govern everything in the chain*. *People need a better understanding on why these issues are important…biosecurity should be number one*, *everything else follows”*. (KII, Biomedical researcher, Kenya)

*“Managing waste is our biggest challenge as it is very expensive*. *Getting the license to allow us to direct our liquid waste to the sewer line costs 100*,*000ksh per year and the officers must come to confirm the viability of the place”*. (Rapid ethnography, Market Manager, Kenya)

*“Street vendors and sellers at temporary markets do not have to pay taxes and official fees*, *their goods are not of quality and clear origin*, *so they sell cheaply*. *The local authorities do not want to close these temporary markets because it is a source of pocket income for them”* (Rapid ethnography, seller, Vietnam)

We found strong sentiments that government support was infrequent and lacking. Food safety was only one part of infrastructure and procedural management, nestled within environmental sanitation, alongside fire prevention and security. The management style of a market also influenced rule and regulation enforcement.

*“A government minister once closed all the slaughter houses here for almost a year because of poor liquid waste management*. *Pretreatment is very expensive and meeting the standards are very hard*. *The construction of a pretreatment firm requires a large space and modern technology*. *The government has not helped us”*. (Rapid ethnography, Slaughterhouse manager, Kenya)

*“In 1993*, *there was a devolution*, *so most of our functions were devolved to [local government]*. *Since at that time*, *[local government] is overwhelmed*, *until now the support towards the agriculture side is very weak…a lot of provinces still do not have a provincial veterinarian*.*”* (KII, Government official, Philippines)

*“[This] is the largest wholesale poultry market in the North*, *built in 1993*, *nearly 30 years now*. *The market facilities have deteriorated*. *The water supply and sewage systems have deteriorated*. *We have proposed to invest in upgrading*, *renovating*, *and expanding the market*, *but so far there have been no plans*.*”* (KII, Market manager, Vietnam)

These challenges were also applicable to markets run by the public sector, where funds for reforms had to be obtained through difficult to obtain centralized government budgets. Infrastructure reform was different for the informal sector due to the temporal nature of these markets, which are usually small and mobile.

### Space and cleanliness

**Fig 5:**
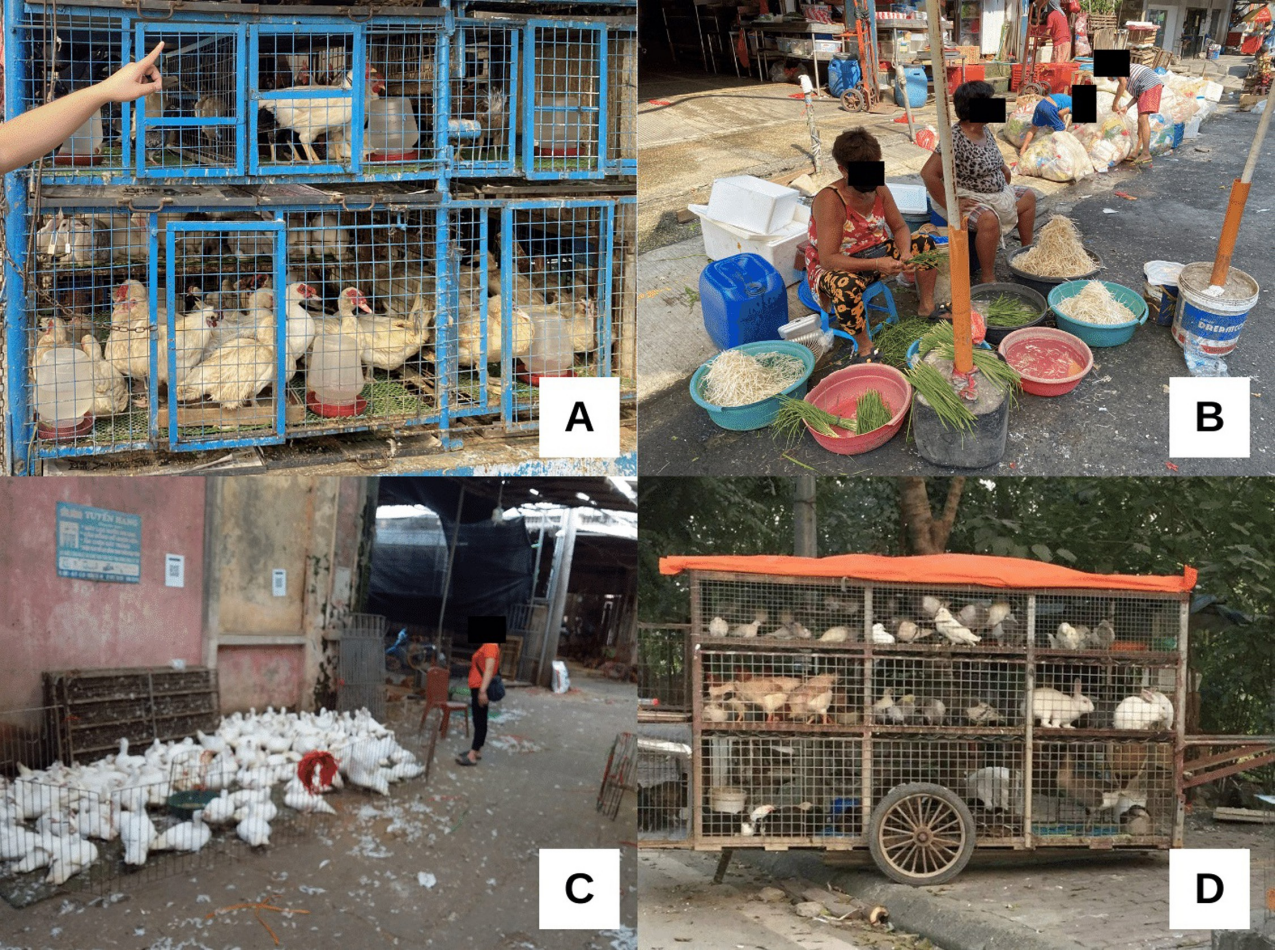
Photos of wet market vendors and animal storage: The Philippines (A/B) and Vietnam (C/D).

The modernization narrative also emphasized a different set of primary concerns about risk. Market hygiene was conceptualized through notions of cleanliness and space (See **Fig 5**), in reference to geographical location. Remote, rural markets were infrequently targetted with infrastructural reforms and biosecurity improvements compared to urban centers.

The cleanliness of markets was widely noted to have improved over the last few decades. This was frequently discussed in reference to spatial and building renovations: spacing of stalls, cemented floors, pulley systems, refrigeration, and better security. Improvements in the physical aesthetics of a market was believed to precipitate a culture of cleanliness with regular routines for sweeping, garbage disposal, and, in urban markets, disinfection (spraying chemicals, soaping and scrubbing). When asked what improvements they would prioritize, most local market stakeholders emphasized building and spatial reorganizations as the main priority. In our urban market site in Manila, Philippines, privatizing the market was associated with building renovations and improved inspection and food safety rules. In Vietnam, newly constructed markets prioritized hygiene rules that separated aquatic, poultry, meat, vegetable and slaughter areas. In Busia, Kenya, new markets had recently been created away from residential areas with fences to keep out dogs.

## Narrative 3: Local livelihoods, restrictions and politics

A third and final narrative centered on how local livelihoods and socio-economic practices conflicted with the goals of biosecurity and modernization. This called into question the appropriateness of biosecurity measures by highlighting punitive bans and policies, the importance of cultural heritage and conflicts between local politicians and veterinary public health.

### The appropriateness of biosecurity measures

Certain biosecurity laws and measures were considered to be inappropriate given local socio-economic conditions. A list of policies mentioned by key informants is provided in **Table 7** Many country policies had been revised after the Avian Influenza outbreak in mid-2000s. Market stakeholders viewed many as a hindrance and annoyance, while veterinary inspectors acknowledged that some were not implementable due to gaps in infrastructure. According to government informants, lack of funding and the fragmentation in coordination mechanisms between local departments, agencies and organizations was the main problem. For example, on-site slaughter is widely practiced in all countries despite it being officially banned in Kenya and Vietnam, creating a legal grey zone.

*“The slaughter of poultry at temporary markets is common…they do not have to pay taxes and fees* . . .*The sanitary conditions are very simple and unsecured*, *but they still do it without any competent management*.*”* (Rapid ethnography, poultry seller, Vietnam)

**Table 7:** Inappropriate policies, noted by key informants*.

Kenya	Vietnam	Philippines
Commercial chicken farmers must have at least five acres of land All livestock (including chickens) should be killed in a slaughterhouse	Veterinary laws ban the sale of live animals at wet markets and require all livestock to be killed at slaughterhouses.	The Local Government Code devolved the regulatory functions of national agencies to the LGU Lower tariffs on imported, frozen pork products

* This is not an exhaustive list of examples.

Slaughter practices prioritize speed over protective wear; infection control material is not available and slaughtering and processing meat is done in tropical temperatures. In the Philippines, we observed many workers without T-shirts, wearing jewelry such as metal bracelets and rings, and vendors moving freely between exchanging money and handling fresh meat with bare hands despite personal protective equipment policies in place. In Nairobi, workers did not like to wear gloves: *“We don’t like using gloves as they are not good to handle meat*, *they are slippery and cause one to work at a slow pace”* (Rapid ethnography, slaughterhouse worker, Kenya). It is difficult to enforce constant hand washing in a busy market, where social norms are guided by ideas of freshness and informal exchange.

Workers also regularly circumvent rules and regulations as part of their business practices. Parts from slaughtered animals are used for different purposes. In the Philippines, pig blood is used in the notable *dinuguan* (blood stew) dish; in Kenya, blood is used for ‘meat sausages’ and several products (bile, omasum, gall bladder stones) are sold on the Chinese export market. In Vietnam, fresh pig or poultry blood can be used to make “*tiết canh*” dishes (blood pudding) without cooking.

*“Frozen meat*, *ideally*, *is prohibited from the wet market*. *If the […] vendor is insistent on selling frozen meat*, *one of the requirements is to have a freezer…[But] you will just see the box is just under the table*, *not in the freezer*. *…It’s still one of our problems*.*”* (KII, Veterinarian, Philippines)

*“Blood that comes directly from the veins of animals is used for making “mutura*.*” We do not recommend people to take blood out of the slaughterhouse [but] people take blood without the meat inspectors consent”* (KII, Meat Inspector, Kenya)

*“After the chickens and ducks are slaughtered*, *I will sell offal to the catfish farmers in the village…Chicken and duck feathers are sold to collectors for processing*. *Wastewater and the rest are poured down the drain*, *from this drain will lead directly to the irrigation ditch in the field*.*”* (Rapid ethnography, Market vendor, Vietnam)

### Punitive and harmful consequences of market rules

The discrepancies between policies on the books and real-world conditions were believed to create ambiguities that contribute to punitive bans and excessive punishments, believed to be harmful for smallholder farmers and the informal sector. In Kenya, this meant that many vendors are not supportive of government policies, and this distrust and conflict was believed to have negative effects on food safety. While some civil society groups (e.g., World Animal Protection) do conduct consumer education about food safety, there appears to be little education and engagement with market stakeholders. Kenyan stakeholders believed that this reduced compliance and drove clandestine activities in the livestock value chain.

*“Unfortunately*, *some of the strategies that come out are “lets ban what the vendors are doing” without providing practical alternatives…This is the mindset we need to change so that we don’t focus only on the law but on incentives*. *e*.*g*. *the poultry farmers slaughter at night when there are no inspectors and then they transport the meat at 3am to the markets*. *So we will continue to play that hide and seek game”*. (KII, International organization, Kenya)

This included the clandestine sale of wildlife meat (zebra and impala in Nairobi) mostly sold in informal settlements and related to poverty. In the Philippines, the wildlife trade at Rio Tuba is similarly conducted in “the shadows” and vendors reported hiding wild boar meat. Poachers receive orders from their “*parokyano”* (loyal customers) or consortia by middlemen, and use social media (ex. Facebook’s Marketplace feature).

*“Although you don’t see it in the wet markets of Palawan*, *it is happening*. *It is happening by text*, *by cellphone*, *by word of mouth within the province*.*”* (KII, Civil society group, Philippines)

*“Wet markets are not places to sell wildlife meat* [but] *people still purchase it because it is cheaper…as long as the demand is there people will find a way to sell their wild meat*. *People will communicate and do it in the black market*. *Health risks are even increased when people go underground”*. (KII, Researcher Kenya)

### Threats to market culture and competition

Market renovations precipitated anxieties, fears and uncertainty about the future and emerged alongside new malls and supermarkets in the urban landscape that compete with the traditional market economy. In this way, modernization was seen by some as a threat to markets as embodied manifestations of cultural heritage. Markets have a unique role in social life where culture (exchanges of recipes, health advice, religious practice) is preserved and preformed; they are sites for jobs, the exchange of money and friendships often located at the historic center of a town, city or village. These sentiments were invoked to explain the threat of modernization, which drew customers away and threatened the traditional social bonds that linked vendors to their clients and supply chains. There were fears about nepotism and monopolies through market relocations and renovations, especially in stall allocations for new markets. New technologies, such as automated systems and digital payments, were also discussed as offering new opportunities to reduce contact between carcasses, customers and workers in biosecurity terms but there was also an acknowledgement that technology could exclude some market stakeholders and impose greater barriers for the informal sector to earn a living by occupying boundary areas outside current surveillance and monitoring.

### Local collective action and political forces

Market vendors noted that they used various strategies of local collective action to protect their interests. For example, during the an informant interview, one market manager mentioned that, in Batangas City, Philippines, they prohibited the sale of stalls to individuals with the intention of renting them to avoid middlemen dominating rental opportunities. Our fieldwork in Kenya found that traders sometimes organize themselves to address market cleanliness and hygiene to avoid complains from the central government.

Wet market reforms intersected with local political interests. Because people, especially ordinary citizens, congregate in wet markets on a regular basis, politicians vying for positions of power and leadership will visit them. For example, in the Philippines a local politician adopted the moniker “Mr. *Palengke*” (Mr. Marketplace). Politicians often control the budget allocations and new mayors were mentioned as turning points in the cleanliness of a market. Local politicians can also interfere with efforts to close an unhygienic market to gain support from the public. In Nairobi, for example, there have been plans to build a modern poultry slaughterhouse since 2015 and this has not happened due to bureaucracy, funds mismanagement and a lack of proper coordination across sectors. The constant changes occasioned by political interference of county officials in Nairobi also contributed to these delays as every officer wants to start his or her own project for political mileage. Politicians may also have an interest in maintaining the status quo and in opposing market closures directed by veterinary disease policy and regulations. In some counties in Kenya, veterinarians have ordered the closure of slaughterhouses due to a disease outbreak only to have county elected officials reopen them to gain political clout and address popular anger and concern about livelihood and food security.

## Discussion

Our study found three competing social narratives that shape biosecurity reforms at wet markets in Kenya, the Philippines and Vietnam: health risks, modernization and local livelihoods. Appreciating how and why social groups hold and experience these narratives differently has important implications for the future of biosecurity investments and policy.

Our project was initially developed to understand the implications of rhetoric in early 2020 calling for the banning and rapid reform of wet markets. This was framed as an urgent priority to “prevent pandemics” [1]. Yet we found no evidence that these calls had any impact in our three case study countries. This is not surprising since many governments (e.g., Vietnam and the Philippines) continued to maintain strict non-pharmaceutical interventions and scaled-up large vaccination programs starting in mid-2021. Greater acceptance of a possible lab origin to the pandemic also drew attention away from an exclusive focus on wet markets [12].

However there are other reasons. Our fieldwork found strong discordance between the bio-securitized framing of wet markets as ‘petri dishes’ of novel viruses in need of urgent sanitization reform, even bans and closures, and the everyday lived experience and cultural meanings associated with them. Universal calls to eliminate wet markets homogenize what are diverse socio-economic systems used by billions of people around the world and also distract from real solutions to multifaceted problems in the agricultural sector [13,14]. Wet markets are overwhelmingly viewed not as risky places that *may* cause a ‘global’ pandemic but as sources of *local* health, food, livelihood and social connection. We found that health concerns were expressed, but in terms of meat safety and food-borne disease. This was related mostly to gaps in basic infrastructure that prevent sanitized slaughter and food hygiene. As found with other studies [7,15], health risks were evaluated based on notions of *freshness* and related to inspection timestamps, on-site slaughter, local production, color and texture, and past food consumption experience.

Rather than viewing markets as sites of novel pathogen emergence, government officials and national scientists were more concerned about local endemic food-borne diseases and trans-boundary animal diseases such as African swine fever and Avian influenza. This echoes findings from Scoones [16], Hinchliffe et al. [17], Bardosh [18], Mwacalimba and Green [19] and others who have argued that international agencies over-emphasize pandemic concerns at the expense of other endemic health priorities. At a country level, national media also appear to over-emphasize the fear of global health crises whereas local health issues appear to receive less attention. In our study, policies and capacity to improve local disease priorities in the animal sector were seen as being stymied by fragmentation and lack of funds but also confusion about specific priorities and the role of different agencies. Similar findings have emerged from other policy analysis studies [20–22]. Key informants called for ‘more One Health’ but it was not clear what this meant in practical terms. One recommendation of note was for more integrated surveillance to detect endemic pathogens of local public health importance.

Those advocating for wet market bans and reforms as a means of pandemic risk mitigation do articulate specific risk practices that should be targeted—related to animal species, hygiene conditions, length of the value chain, etc. [6,21]. The term ‘wet market’ is a heterogeneous category [6]; diversity is found within and between different markets, areas of a single market and different stakeholders across the formal and informal value chain. We encourage researchers and local government to conduct country-specific evaluations of wet markets conditions and biosecurity practices to better appreciate the diversity of local markets, and to use this contextualized information to better understand gaps and plan for feasible and desirable improvements.

There continues to be debate about the need, feasibility and consequences of wildlife trade bans for nature conservation and pandemic prevention [4,11,23]. Our research was exploratory and had only a limited focus on the wildlife trade; however our data suggests that reform efforts to limit exotic animals at formal markets have taken place over the last few decades and consumers are also more aware of existing wildlife laws. This suggests that the global rhetoric about the dangers of the wildlife trade and the need for blanket policies for pandemic risk may be overstated. From a novel pathogen standpoint, there is a need to better define ‘high-risk activities’, anticipate the unintended consequences of stricter biosecurity reforms and consider what types of risk mitigations are really possible given the inherent uncertainty in predicting disease dynamics for a low probability ‘pandemic’ event.

Perhaps contrary to what we expected according to global rhetoric, we found that wildlife rarely ends up being openly sold at many markets. Some of our study locations were well-known nodes in the live wildlife trade but we observed very few wild animals available for sale. Clandestine sales are reported to occur however, and we sampled a small number of markets. The Covid pandemic may also have changed existing trade networks. It was clear, however, that a transition is occurring from catching wildlife in the wild to a growing intensive exotic animal farming industry, especially in Vietnam but also in the Philippines, with limited controls and law and veterinary oversight [23]. This should be prioritized more in infections disease risk research efforts.

We found two concepts particularly important across the three countries. *Freshness* was the dominant conceptual lens by which market stakeholders viewed and interpreted health whereas *cleanliness* was the dominant lens used to understand infrastructural improvement. In this sense, *cleanliness* helped ensure *freshness*. Markets reflected a local process of historical and socio-economic modernization and we found that most had undergone recent improvements in slaughter, transport and waste collection. The framing of markets as sites of *modernization* emphasized a different set of primary concerns about risk related to security and economy compared to risk in biological and health terms. Market hygiene was conceptualized through notions of cleanliness and space, both of which were linked to the geographical location and physical characteristics of a market [24]. Improving biosecurity was inseparable from general infrastructure and management improvements and were mediated by costs and political will.

When asked what improvements they would prioritize, most local market stakeholders emphasized building and spatial reorganizations as the main priority. This finding fits within some specific recommendations made by organizations calling for the separation of wildlife and livestock as part of a pandemic prevention strategy [6]. However, the history of past market reforms, which occur in ways that are not always favorable to local stakeholders, should provide a degree of caution as rapidly organized interventions cause negative consequences and are not sustainable. Many past reforms involve moving markets out of crowded traditional areas and come with fences, security, new stalls and cement–all of which make markets look and feel more like a supermarket. Our study showed how modernization–viewed as “both a blessing and a curse”–was also associated with the consolidation of economic power and deterioration in cultural heritage and social bonds. Market vendors and sellers expressed anxieties about an uncertain future as new shopping malls and supermarkets continue to expand in the urban landscape. Although open-air markets are predicted to continue to be a main source of food, including in urban Africa [25], this conflict will accelerate in the coming years [26]. Efforts should be made to ensure the cultural preservation of traditional wet markets.

Markets are sites of conflict between the interests of local market sellers and traders and the goals of biosecurity and economic modernization. One important issue we found involved laws and regulations banning animal slaughter outside official slaughterhouses in Vietnam and Kenya that emerged in the aftermath of the Avian Influenza crisis in the mid-2000s [27,28]. The discrepancy between these policies and real world conditions and needs create ambiguities that were believed to contribute to punitive bans and excessive punishments implemented inconsistently at the local level. A large literature has explored the unintended consequences of biosecurity laws and enforcement in the livestock sector, questioning this type of heavy-handed approach [27,29]. And yet there is often a minimization of the biosecurity risks associated with modernized and sanitized market actors as well as an under-appreciation of how local practices in smaller-scale systems may reduce risk [27,30,31]. One result of this is an increase in animosity and distrust between vendors (concerned with securing their livelihoods) and veterinary and market authorities (concerned with increasing biosecurity). There is some evidence this may be counter-productive as it reduces support for government policies and may drive clandestine activities in the livestock value chain.

Finally, we found that conflicts at markets do elicit forms of popular political action, in efforts at self-organization (e.g., to clean markets and maintain payment fees) and to resist specific policies (e.g., animal movement bans and closures). In the context of decentralization reforms, present in all three case study countries, local government plays an increasingly pivotal role in market management and regulation for services and facilities. Ensuring the *health* of local food systems, of which wet markets play a central role, is part of a broader struggle between policy ideologies in food systems (e.g. local vs. the global, the small-scale vs. the corporate, the fresh vs. the packaged) and manifested in struggles between grassroots activism, government regulations and the economic centralization of power [32,33]. There is a need to better understand how local government can strengthen market management and biosecurity in ways that enhance the agency of market stakeholders and strengthen local livelihoods and food security as part of a pluralistic and democratic politics.

## Study limitations

There are several limitations to our exploratory study. The first involves the short period of time spent at wet markets (1–2 days per site during one visit in Kenya and the Philippines), which limited the depth of certain sensitive topic (e.g., illegal wildlife trading and non-compliance with biosecurity practices). Longer-term ethnographic research is needed to better understand the nature of the market chain, how different types of markets are governed and managed differently and the opinions and experiences of customers. There were specific policy points raised by only one key informant that we did not have sufficient time to clarify (e.g., details of previous biosecurity policies and the nature of the wildlife trade). Fieldwork was also conducted during the Covid-19 pandemic, which impacted the social context of data collection (e.g. requirement to use masks and social distance, and multiple local quarantines in Vietnam). In spite of these limitations, the research team felt that this exploratory study captured sufficiently data for the overall objectives and that our analysis was robust and trustworthy.

## Supporting information

S1 File Ethnography Guide: Rapid ethnography guide. (DOCX)

S2 File Interview Guide: Key informant interview guide. (DOCX)

S3 File Inclusivity Questionnaire: Inclusivity in global research questionnaire. (DOCX)

S4 File Stakeholder Map: Kenya stakeholder map. (TIFF)

S5 File Stakeholder Map: Philippines stakeholder map. (TIFF)

S6 File Stakeholder Map: Vietnam stakeholder map. (TIFF)
