# Correction: Wet market biosecurity reform: Three social narratives influence stakeholder responses in Vietnam, Kenya, and the Philippines

**DOI:** 10.1371/journal.pgph.0002859

**Published:** 2024-01-18

**Authors:** Kevin Bardosh, Renzo R. Guinto, Salome A. Bukachi, Tran Minh Hang, Marianne K. Bongcac, Mara Ysabella M. de los Santos, Caroline M. Mburu, Jackielyn Abela, David Kelly, Cecily Maller

A panel was erroneously left out of Fig 2. Please see the revised Fig 2 below.

**Fig 2:**
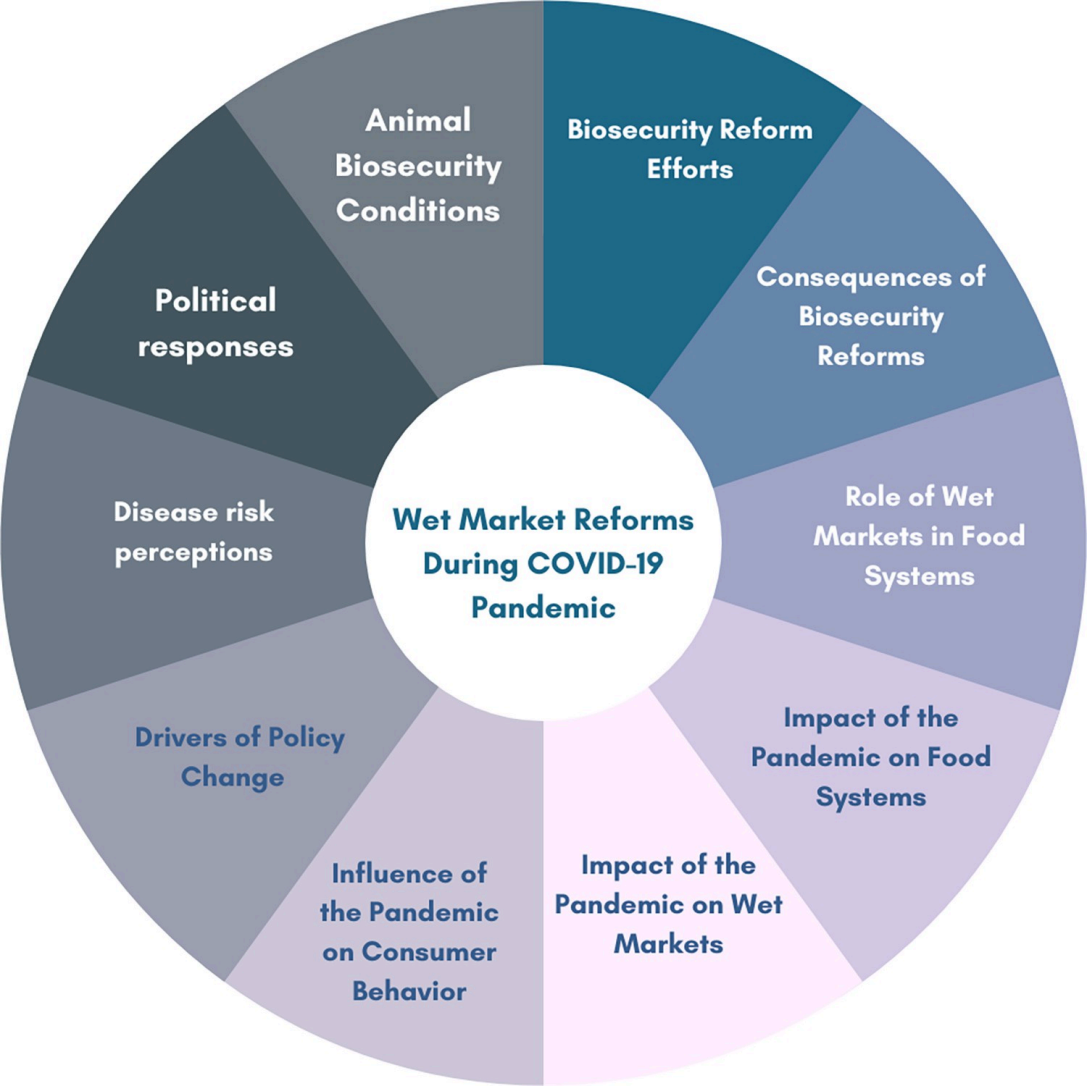
Ten main themes used in the final analysis of fieldwork data.
