# Supplementary material for: Wet market biosecurity reform: Three social narratives influence stakeholder responses in Vietnam, Kenya, and the Philippines
Source: PLOS Glob Public Health. 2023 Sep 6;3(9):e0001704. doi: 10.1371/journal.pgph.0001704 (PMC10482282; doi:10.1371/journal.pgph.0001704)
Supplement: S1 File — (DOCX) [file pgph.0001704.s001.docx]

**Rapid ethnographic wet market fieldwork guide**

Rapid ethnography involves the use of ethnographic methods focused on specific issues and conducted over a short period of time. In our project, we will use this approach during visits to 2-3 wet markets in each case study country.

**Fieldwork guidelines**

Each team should select 3 markets from the initial list of 10 markets. Selection should be discussed with all members of the project team. We would like to concentrate on markets that pose a potential risk for disease emergence, which means that those selling live animals and wildlife should be prioritized.

Each market should be visited for 2-4 full days (depending on logistics).

Our approach to fieldwork will be didactic. The research team should aim to interview as many different types of people as possible in order to build a conceptual interpretation of the major thematic areas of interest. The team should use the following methods:

1. Unstructured and semi-structured interviews
2. Informal group conversations
3. Transect walks
4. Direct observations
5. Participant observation

The 10 thematic areas (listed below) are the main topics of interest for our research at wet markets. Research teams should develop their own strategy to fieldwork at the markets following this list of themes and questions. Under each thematic area there is a list of research questions that we would like to answer. These questions should be adapted before they are asked to each research participant at the market. The research team is responsible for adapting these questions in appropriate ways. This will require preparing before the fieldwork period.

Interviews and observations at the wet markets should be focused on 1 thematic area at a time. This will make the fieldwork manageable for the researchers. Researchers can also divide the thematic areas and address them during different visits to the market sites.

It is recommended that researchers print out each thematic area onto a separate piece of paper so that it is easy to walk around the market and conduct fieldwork. Interview questions can be written on this paper. This should be used to help structure interviews. Notes should also be arranged by thematic area to make it easy to transcribe.

Sampling should also be dynamic. We should aim to combine opportunistic and purposive sampling. An important part of our approach will be to triangulate findings across different social groups. In this regard, we would like to understand social difference at wet markets and how these differences influence perceptions and practices. The major category of informants we would like to engage include:

1. Animal traders
2. Butchers
3. Vendors
4. Consumers
5. Market managers
6. Veterinary inspectors

**Data collection**

Capturing data is a challenging task in rapid ethnographic fieldwork. We will not be using an audio recorder at wet markets but will rely solely on note taking. A few high quality photographs should also be taken from an iPhone. Each researcher should carry two notebooks: one small pocket-sized notebooks to record observations and key ideas during informal conversations as researchers walk around the market and a larger notebook to record longer interviews and group discussions conducted while sitting down.

Notes should also be arranged by thematic area to make it easy to transcribe.

When taking notes, researcher should aim to:

- Retain the informants’ exact words, as closely as you can. This is especially important for key phrases or creative sayings that the informant used to make an important point.
- For very important facts, you can ask: “wait a minute, this is very important for me to write down as you have just said it.” Do this only for very important facts. You can read back your notes to the informant and ask them if you missed anything.
- After each discussion, or right before it ends, record the general profile of the participants: gender, estimated age and type of informant (position)
- At the end of each research encounter, researchers should review their notes and add important information that the participant(s) said which might have been missed during the interview.

Researchers should discuss their findings as they generate data in order to build a conceptual understanding of the thematic areas. During each fieldwork day at the market, researchers should review their notes at lunchtime to make a plan for what data is missing and needs to be followed-up.

After each market fieldwork day, researchers should spend 1-2 days transferring all of their notes to a Microsoft Word document. This document should follow the following format:

1. Details about the fieldwork day.
2. Different types of research methods used and number of participants in each method. A table should be created to display these details.
3. Research findings should be organized following the 10 thematic areas. Notes should be written below each thematic area. Direct quotes of important statements and key high-quality photographs should be included. Text boxes can also be used to illustrate important examples or in-depth analysis. For quotations, it is important to maintain the details of the participant. For example: (Female, 35 years old, butcher, Nairobi central market)
   1. **General market characteristics**
   2. **Animal supply chains**
   3. **Animal biosecurity conditions**
   4. **Perceptions of disease risk at markets**
   5. **Consumer preferences**
   6. **Pandemic restrictions and the market**
   7. **The impact of the pandemic on food systems**
   8. **The impact of the pandemic on the wildlife trade**
   9. **Management of the market**
   10. **Change at the market**
4. Reflections on the fieldwork day and a list of next steps for follow-up visits to the market.

**Final report**

For the final report of the rapid ethnographic research, each individual market visit data transcript should be synthesized together into one document following the same thematic categories and format. This report should discuss differences and similarities between market sites.

##

**Rapid ethnographic research: 10 thematic areas**

**1. General market characteristics**

*This section is a minor part of the research but should be asked to generate background information.*

- 1. What is the name of the market; when is it open?
  2. How old is the market? How large is the market?
  3. How many customers come to the market and how does this change with the seasons?
  4. What goods are sold at the market?
  5. How many vendors (roughly) are there at the market?
  6. How many vendors (roughly) are involved in the sale of animal meat?
  7. What is the average income of those working in the animal meat sector at the market?
  8. What vendor fees are collected at the market?
  9. Is the market crowded?
  10. What types of people come to the market? Are certain ethnic or socioeconomic groups more likely to come to the market? If so, why?
  11. What positive things do people say about the market?
  12. What negative things do people say about the market?

**2. Animal supply chains**

*This section should be accompanied with observational notes and, if possible, photographs of the different types of live animals sold at the market.*

- 1. What types of live animals are sold at the market?
  2. Where do the animals come from? Do they come from large or small farms?
  3. How are the animals transported to the market?
  4. How long can a live animal be stored at the market?
  5. If animals are not sold at this market, are they moved to another market to be sold?
  6. If wildlife is sold at the market, are they wild caught or bred in captivity?
  7. Are there any endangered/threatened wildlife sold at the market?
  8. Are any animal parts sold as medicine at the market? If so, what types?

**3. Animal biosecurity conditions**

*This section should be accompanied with observational notes and, if possible, photographs of biosecurity conditions and practices.*

- 1. How are the live animals caged and stored at the market? Are different species of animals mixed in the same cage? Are the cages crowded? How close are cages from different animal species?
  2. Are the cages hygienic and regularly cleaned?
  3. What do workers think about the mixing of animals and the hygiene conditions at the market?
  4. How are animals slaughtered at the markets?
  5. Do workers use any protective gear when slaughtering and handling animals?
  6. Is there water and washing stations for the workers?
  7. How are animal waste products disposed of at the markets?
  8. How are markets cleaned?
  9. What risky practices do you observe?
  10. How have workers tried to improve biosecurity at the market?
  11. Does a lack of infrastructure at the market prevent certain biosecurity measures from being implemented?
  12. How has the pandemic changed biosecurity measures and practices for animals at the market?

**4. Perceptions of disease risk at markets**

- 1. What types of disease risks do people associate with working at the markets?
  2. What types of disease risks do people associate with buying animals or meat at the markets?
  3. Has the market ever been a source for a disease outbreak in the past? If so, what are the details?
  4. Has the market ever been shutdown due to a disease outbreak before?
  5. Do people think this wet market could help a new disease to emerge from animals to the human population? If so, why and how?
  6. How has the pandemic changed people’s perception of disease risk from wet markets?
  7. What do people think caused the COVID-19 pandemic?
  8. Do people have a more negative perception of wet markets as a place where new diseases may emerge due to the COVID-19 pandemic?

**5. Consumer preferences**

5.1. Why do people prefer to buy their meat at the market and not somewhere else?

5.2. Do people think it is healthier to obtain their meat from the markets compared to supermarkets?

**6. Pandemic restrictions and the market**

- 1. What types of restrictions have been placed on wet markets during the pandemic?
  2. What do people think about the pandemic restrictions at wet markets?
  3. Were there calls from the government or media to ban or shut wet markets in the country?

**7. The impact of the pandemic on food systems**

- 1. How did the pandemic restrictions at the market affect access to food?
  2. How did the pandemic restrictions at the market affect smallholder farmers?
  3. How did the pandemic restrictions at the market affect workers and vendors?
  4. Did the pandemic restrictions at the market disproportionately affect certain people (women, farmers, the poor) who depend on wet markets?
  5. Were pandemic restrictions imposed equally across the food system (both at supermarkets vs wet markets)?
  6. Has the pandemic led to people increasing their shopping at supermarkets and online vendors? Do people think this trend will continue into the future?
  7. Did the pandemic reveal food system vulnerabilities?
  8. How were food prices, specifically of animal protein, affected by the pandemic?
  9. Has the pandemic equally affected smallholder livestock farmers and larger agribusinesses?
  10. Has the pandemic affected the formal and informal sector differently?
  11. Has anyone in the food sector benefited from the crisis?

**8. The impact of the pandemic on the wildlife trade**

- 1. How has the pandemic affected the wildlife trade?
  2. Has the pandemic increased or decreased the hunting and sale of wildlife?
  3. Has the pandemic increased or decreased the rearing of wild animals?
  4. Has the pandemic led to new policies and efforts to control the wildlife trade?

**9. Management of the market**

- 1. What are some of the major management challenges with the market?
  2. What infrastructure upgrades are planned or needed?
  3. Who is in charge of biosecurity measures at the market? What activities do they do?
  4. What types of regulations and laws related to animals do the market managers and veterinary staff know about?
  5. Do people think that the biosecurity regulations and laws are enforced?
  6. Do people think that the biosecurity regulations and laws are reasonable?
  7. Are some of the biosecurity measures ignored?
  8. What laws and regulations are not enforced or hard to enforce?
  9. Have there ever been any protests from vendors and workers about enforcement of laws at the market?
  10. Have there ever been any protests from vendors and workers about working conditions?

**10. Change at the market**

*This section should discuss broader change dynamics at wet markets and how these forces have influenced and will influence biosecurity, animal issues and sale of animal products*

- 1. What changes have taken place in the market over the last 20 years?
  2. What changes have been good?
  3. What changes have been bad?
  4. Why did these changes occur? Were they caused by local or national government policy? Were they caused by socio-economic changes?
  5. Who wanted these changes and why?
  6. Were some people upset with these changes?
  7. In the next 10 years, how do people think the market is going to change?
  8. How can biosecurity be improved at the market?
  9. How can the market be improved in general?
