# Supplementary material for: Wet market biosecurity reform: Three social narratives influence stakeholder responses in Vietnam, Kenya, and the Philippines
Source: PLOS Glob Public Health. 2023 Sep 6;3(9):e0001704. doi: 10.1371/journal.pgph.0001704 (PMC10482282; doi:10.1371/journal.pgph.0001704)
Supplement: S3 File — (DOCX) [file pgph.0001704.s003.docx]

**Interview guide 1: Pandemic risk from wet markets**

The aim of these interviews is to explore how the pandemic has affected the framing and perception of disease risk from wet markets as well as biosecurity efforts to address them. These questions should be adapted based on the particular stakeholder you are interviewing.

Start: *Thank you for agreeing to participate in this study. To get started, can you please tell me a little bit about yourself and how your current work relates to wet markets?*

1. **Before COVID-19, were wet markets seen as a source of emerging disease and pandemics?**
   1. Was this considered a priority issue?
   2. Was the risk associated with certain types of wet markets or areas of the country?
   3. What types of market practices were seen as the most important or the most risky?
   4. What emerging pathogens were considered the main priorities?
2. **Before COVID-19, what activities were taking place in [country] to address the risk of pandemics at wet markets?**
   1. Were you personally involved in any of these activities?
   2. Possible areas to mention: Surveillance; scientific research; biosecurity; infrastructure; WASH interventions; capacity building in the animal/veterinary sector; food safety; One Health; medical countermeasures.
3. **Apart from emerging diseases, wet markets are also associated with food-borne, ARM and endemic zoonotic diseases. Are these seen as a priority by the health sector?**
   1. What diseases are prioritized specifically?
   2. What types of programs exist to address them?
4. **What are some of the strengths and weaknesses that you have observed with efforts to improve biosecurity at wet markets in the country?**
   1. What lessons were learnt in past outbreaks? (i.e. avian influenza)
   2. Is there enough collaboration between animal and human health sectors?
   3. What challenges have you observed with the design /implementation of policy?

*So far, we have been discussing health risks and biosecurity at wet markets before the pandemic. Now I would like to ask some questions about how the pandemic has affected things.*

1. **Since the COVID-19 pandemic began, some global organizations have been very focused on the question of disease emergence from wet markets around the world. Did this have any influence on wet markets in the country?**
   1. Did certain organizations call for the closure or banning of wet markets?
   2. If so, who was saying this and why? (i.e. specific organizations)
   3. If so, was the call targeted to some specific types of wet markets or to all?
   4. Did it take place and, if so, was there any resistance to this?
   5. Do you think this approach was based on science or evidence?
2. **Do you think the pandemic has changed people’s perception of disease risk from wet markets in any way?**
   1. Did markets and wild animals suddenly become perceived as bad or dangerous?
   2. Did consumers become hesitant to go to wet markets, for example to buy meat?
   3. What role did the media play in framing and amplifying the issue?
   4. Have these perceptions changed and evolved over the course of the pandemic?
3. **How has the pandemic changed wet market policies and biosecurity?**
   1. Possible areas to mention: Surveillance; scientific research; biosecurity; infrastructure; WASH interventions; capacity building in the animal/veterinary sector; food safety; One Health; medical countermeasures.
   2. Have all of these changes been good or effective?
   3. Have some stakeholders been excluded from the decision-making process?
4. **Do you think wet markets in [country] are really a major risk for the emergence of a global pandemic?**
   1. Do you think we have a good scientific understanding of spillover risk from wet markets?
   2. What important gaps in understanding or knowledge exist?
   3. Do you think the risk has been overblown?
5. **In your opinion, who has been guiding the agenda as it relates to wet markets and pandemic risk?**
   1. International agencies? Health sector? Conservation sector? Central government? Defense agencies? Scientists?
   2. Are some stakeholders being excluded from the decision-making process?
   3. Do you think that international actors have had too much influence on national policy priorities and decisions in this area?
   4. Do you think the focus on addressing pandemic risk negatively impacts the effort to control endemic diseases?
6. **As you know, wet markets are, in many cases, the end of the value chain for the trade in wildlife (live and dead exotic animals). And this trade also presents, according to some, a degree of risk for the emergence of pandemics. How has COVID-19 affected the wildlife trade?**
   1. Do you think these changes have increased or decreased the health risks associated with wet markets?
   2. Do you think these changes are being accounted for in policies and programs?
7. **Thinking into the future, say 5-10 years from now, what major changes related to wet markets do you anticipate will happen?**
   1. How do you think these will influence health risks?
   2. Who will be most important in pushing these reforms forward?
   3. How can policies and reforms be improved?

**Interview guide 2: The effects of the pandemic on wet markets**

The aim of these interviews is to explore how the pandemic has affected the functioning of wet markets, including socio-cultural, nutritional, economic and physical dimensions. These questions should be adapted based on the particular stakeholder you are interviewing. Remember that we are most interested in issues related to animals at wet markets.

Start: *Thank you for agreeing to participate in this study. To get started, can you please tell me a little bit about yourself and how your current work relates to wet markets?*

1. **How important are wet markets to the food system in [country]?**
   1. How are wet markets involved in animal value chains?
   2. Are certain types of animal products predominately sold at wet markets?
2. **In what ways do you think wet markets are important for the economy and society?**
   1. Are wet markets important for certain types of consumers?
   2. Besides food, why else do people go to wet markets?
   3. How about certain types of social groups in terms of employment?
   4. How about certain types of farmers or fisherfolk?
3. **How have wet markets changed over the last 20 years?**
   1. What changes have been good?
   2. What changes have been bad?
   3. What are the most important drivers for these changes?
4. **What key policies have influenced wet markets over the last few decades?**
   1. For example: policies related to biosecurity, infrastructure, rural development, food systems, health, and government administration.
   2. Has there been resistance from market vendors and consumers to these policies?
   3. Do you think these policies have always been appropriate and implemented effectively?
   4. How have these policies affected the cultural and social value of wet markets?
5. **Who are the key policy actors that have shaped reform policies at wet markets?**
   1. Which actors have the most power and authority?
   2. Who has the least authority and power?
   3. What types of conflicts and tensions exist between the different stakeholders?
   4. How have compromises between these groups been reached in the past?

*So far, we have been discussing the economic and livelihood importance of wet markets. Now I would like to ask some questions about how the pandemic has affected people.*

1. **What types of restrictions have been placed on wet markets during the pandemic?**
   1. Do you think all of these restrictions were necessary?
   2. What do people think and say about the restrictions?
   3. Have restrictions changed over time?
   4. Are all wet markets impacted the same way?
2. **Since the COVID-19 pandemic began, some global organizations have been very focused on the question of disease emergence from wet markets around the world. Did this have any influence on wet markets in the country?**
   1. Did certain organizations call for the closure or banning of wet markets?
   2. If so, who was saying this and why? (i.e. specific organizations)
   3. If so, was the call targeted to some specific types of wet markets or to all?
   4. Did it take place and, if so, was there any resistance to this?
   5. How has this impacted people’s perception of wet markets?
3. **How did the pandemic restrictions at wet markets affect people?**
   1. How did they affect access to food?
   2. How did they affect smallholder farmers?
   3. How did they affect market vendors?
   4. Did they disproportionately affect certain people (women, farmers, the poor) who depend on wet markets?
4. **How has the pandemic revealed food system vulnerabilities?**
   1. How were food prices, specifically of animal protein, affected by the pandemic?
   2. Has the pandemic equally affected smallholder livestock farmers and larger agribusinesses?
   3. Has the pandemic affected the formal and informal sector differently?
   4. Has anyone in the food sector benefited from the crisis?
5. **How has the pandemic changed the food retail and shopping sector?**
   1. Have people increased their shopping at supermarkets and online vendors?
   2. Do you think these trends will continue into the future?
   3. How have wet markets responded to the competition?
6. **We know that some wild animals and meats are sold at some markets. How has the pandemic affected the wildlife trade?**
   1. Has the pandemic increased or decreased the hunting and sale of wildlife?
   2. Has the pandemic increased or decreased the rearing of wild animals?
   3. Has the pandemic led to new policies and efforts to control the wildlife trade?
7. **Thinking into the future, say 5-10 years from now, how do you think consumer patterns at wet markets will change in [country]?**
   1. How can policies and reforms be improved to benefit all people?
