# Supplementary figures and images for: Wet market biosecurity reform: Three social narratives influence stakeholder responses in Vietnam, Kenya, and the Philippines
Source: PLOS Glob Public Health. 2023 Sep 6;3(9):e0001704. doi: 10.1371/journal.pgph.0001704 (PMC10482282; doi:10.1371/journal.pgph.0001704)

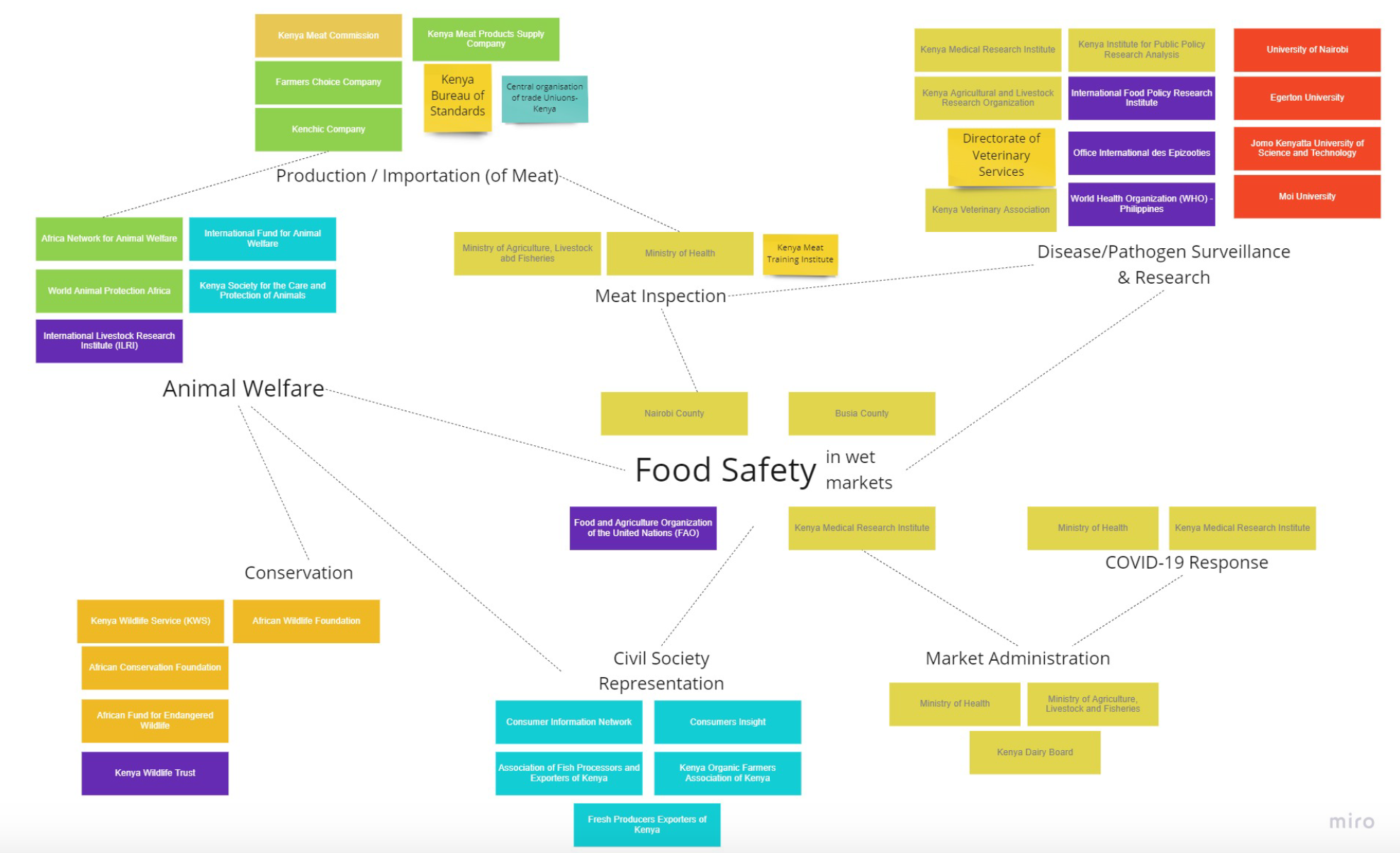

Supplement: S4 File — (TIFF) [file pgph.0001704.s004.tiff]

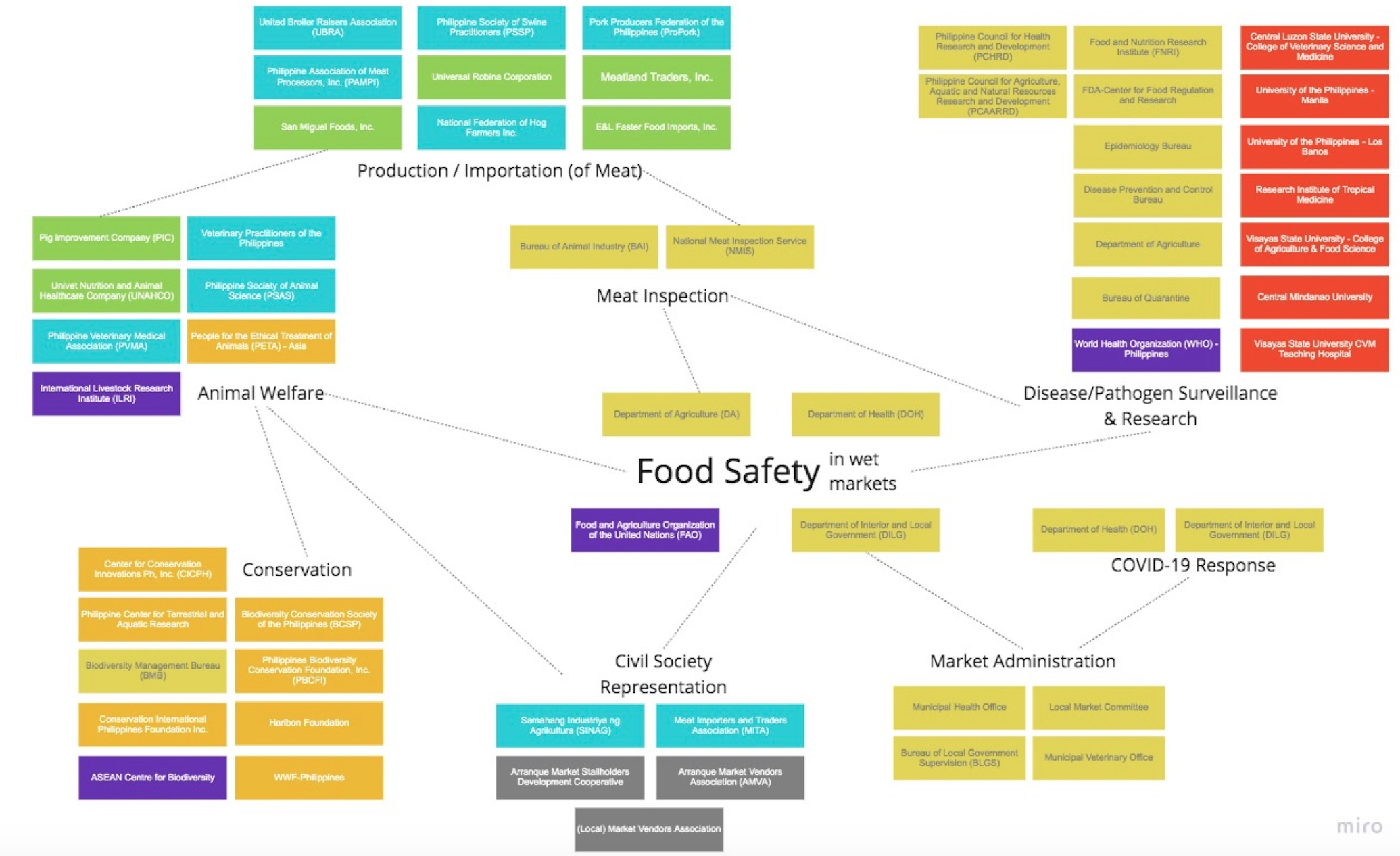

Supplement: S5 File — (TIFF) [file pgph.0001704.s005.tiff]

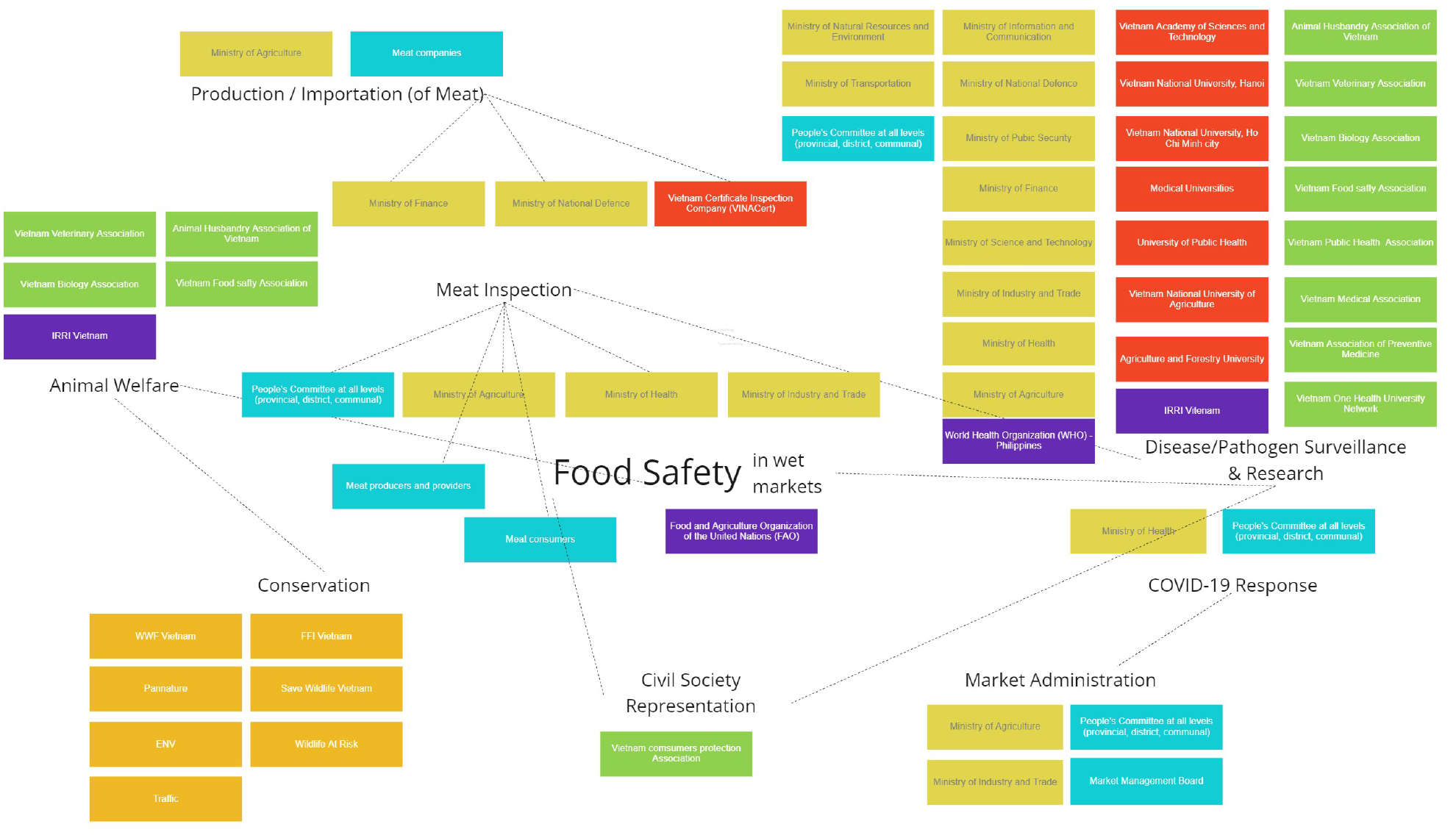

Supplement: S6 File — (TIFF) [file pgph.0001704.s006.tiff]
